# Evolutionary dynamics of a rapidly receding southern range boundary in the threatened California Red‐Legged Frog (*Rana draytonii*)

**DOI:** 10.1111/eva.12067

**Published:** 2013-04-03

**Authors:** Jonathan Q. Richmond, Kelly R. Barr, Adam R. Backlin, Amy G. Vandergast, Robert N. Fisher

**Affiliations:** ^1^ U. S. Geological Survey Western Ecological Research Center San Diego CA USA

**Keywords:** conservation genetics, phylogeography, ranid frogs, range edge ‐ empirical, wildlife management

## Abstract

Populations forming the edge of a species range are often imperiled by isolation and low genetic diversity, with proximity to human population centers being a major determinant of edge stability in modern landscapes. Since the 1960s, the California red‐legged frog (*Rana draytonii*) has undergone extensive declines in heavily urbanized southern California, where the range edge has rapidly contracted northward while shifting its cardinal orientation to an east‐west trending axis. We studied the genetic structure and diversity of these frontline populations, tested for signatures of contemporary disturbance, specifically fire, and attempted to disentangle these signals from demographic events extending deeper into the past. Consistent with the genetic expectations of the ‘abundant‐center’ model, we found that diversity, admixture, and opportunity for random mating increases in populations sampled successively further away from the range boundary. Demographic simulations indicate that bottlenecks in peripheral isolates are associated with processes extending tens to a few hundred generations in the past, despite the demographic collapse of some due to recent fire‐flood events. While the effects of recent disturbance have left little genetic imprint on these populations, they likely contribute to an extinction debt that will lead to continued range contraction unless management intervenes to stall or reverse the process.

## Introduction

A variety of evolutionary and ecological phenomena create greater instability in populations at the edge of a species' distribution compared with those located more toward the interior. These can include maladaptation to environments at or beyond the current boundary, increased competition with other species, reduced immigration due to greater isolation, low genetic diversity, smaller effective population sizes, or interactions between these and other variables (Holt 2003; Angert and Schemske 2005; Eckert et al. 2008; Hardie and Hutchings 2010). When anthropogenic stressors are added to the equation (e.g., urbanization, fires, introduction of non‐native species, pollutants, etc.), persistence at the range boundary becomes even more difficult because the temporal scales over which these stressors operate often do not allow sufficient time for adaptation, particularly for species with long generation times or habitat specialization. This problem can be exacerbated if the range edge interfaces with human population centers, as preferred habitat tends to be more fragmented and dispersal into disturbance‐free areas may not be possible.

Because these phenomena have the potential to reduce the performance of fitness‐related traits and adaptive potential, peripheral populations are controversial targets for conservation (Hunter and Hutchinson 1994; Lesica and Allendorf 1995). On the one hand, their frontline position at the edge of a species distribution can make them more vulnerable to the effects if climate change or other environmental perturbation, particularly if their adaptive potential is stifled by low genetic diversity and restricted migration (Hoffmann and Blows 1994; Blows and Hoffmann 2005). On the other hand, edge populations may also facilitate lineage diversification, so long as sufficient adaptive diversity is maintained in the population, gene flow is limited, and selection is strong (Channell and Lomolino 2000). Thus, in cases where declining peripheral populations of an otherwise widespread species have led to conservation concern, the decision of whether to legislatively protect such species may rest to a large degree on how much diversity has been lost at the range margin, the causes for decline, and whether that loss can be mitigated or reversed through management efforts.

One example of a threatened species with receding range edges is the California red‐legged frog (*Rana draytonii*). Since the 1960s, a gap between extralimital populations in the San Pedro Martir Mountains of Baja California and southern California has been widening (~500 km), with the southern range limit in California contracting northward along the coast (Jennings and Hayes 1994; USFWS 2002 Fellers 2005). The rapid disappearance of *R. draytonii* in southern California, combined with declines in the northern Sierra Nevada foothills and around the north end of the Central Valley (Fisher and Shaffer 1996; Davidson et al. 2001 Fellers 2005), suggests that the species range is shrinking inward toward its core in the central coast of California. Although range edges are inherently dynamic to some degree, the speed and severity of edge decay for *R. draytonii* in the southern part of the state have been linked to a common set of factors involved with the decline of amphibians worldwide (Lannoo 2005; Wake 2012), many of which are directly related to human activity. Of particular relevance to populations in southern California is the ever‐growing urban landscape and the numerous fires that charred large open areas between 2002 and 2010. Several of these fires resulted in severe demographic declines at the range edge, but their effects on genetic diversity, differentiation, and the long‐term survivorship of peripheral populations are not known. In addition to (but not necessarily exclusive to) human‐mediated disturbance, the pattern and speed of edge contraction are consistent with the spread of a fatal pathogen. Due to the recent loss of amphibian diversity worldwide to chytridiomycosis, the disease that often develops from infection by the chytrid fungus *Batrachochytrium dendrobatidis*, it is possible that at least some mortality in southern California could reflect the presence of this pathogen. However, acute die‐offs that characterize most chytridiomycosis*‐*susceptible species have not been observed for *R. draytonii*, and the tendency for members of this species to develop disease in the wake of infection is unclear (but see Padgett‐Flohr 2008; Tatarian and Tatarian 2010).

In this study, we use microsatellites and mtDNA data to examine the evolutionary dynamics of the receding range boundary for *R. draytonii* in southern California and tested hypotheses related to the ‘abundant‐center’ model (Hengeveld and Haeck 1982; Brown 1984; Eckert et al. 2008). This model assumes that individual survival and reproduction are highest under optimal conditions where the species is most abundant and lower in areas that deviate from this optimum. From a spatial perspective, abundance is predicted to decrease from the center toward the range edges, with edge populations being progressively smaller and more isolated (Vucetich and Waite 2003). A simple genetic extension of these predictions is that effective size, genetic diversity, and gene exchange decline toward the range margins (Eckert et al. 2008), thus providing a straightforward set of hypotheses to be tested in this study. In addition to these center‐edge dynamics, we also studied edge‐to‐edge gene exchange due to the possible presence of favorable alleles or gene combinations in the marginal environment (Sexton et al. 2011). In such cases, edge exchange may be preferable to center‐edge flow of nonlocal genes that could potentially accelerate declines or stall niche expansion. Other specific goals of this work include a quantification of the genetic effects of fire in edge populations with known demographic and fire histories, and the development an evolutionary genetic framework for informing reintroduction, and genetic rescue efforts for *R. draytonii* in southern California.

## Materials and methods

### Sampling

**Figure 1:**
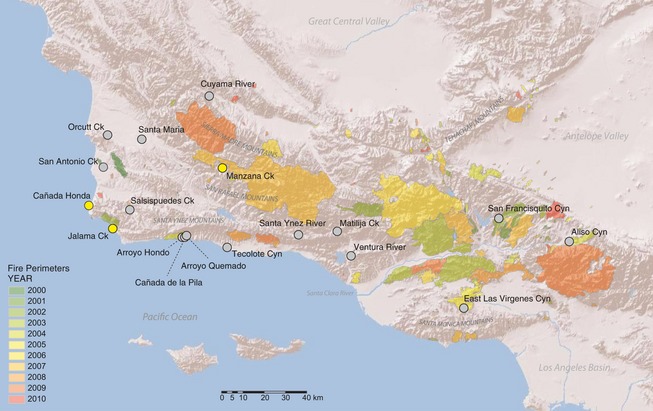
Sampling locations and fire perimeters (2000–2010) in southern California. Sites with yellow bullets have mixed mitochondrial clade membership.

We collected toe clips from all known *R. draytonii* populations in southern California (Fig. 1; Table S1 provides location data and sample sizes). Three populations, San Francisquito, East Las Virgenes, and Aliso Canyons have well‐documented fire histories and census data extending over the past decade: San Francisquito was heavily impacted by the Copper Fire of 2002 (95 km^2^), East Las Virgenes by the Topanga Fire of 2004 (98 km^2^), and Aliso by the Crown Complex Fire of 2004 (73 km^2^) and the Station Fire of 2009 (650 km^2^; www.fire.ca.gov). Sampling at San Francisquito consisted of a temporal series, one representing a preburn group (collected in 2002) and two representing postburn groups (2005/06 and 2009). We combined the 2005–06 cohorts due to small sample sizes and because both were sampled after anomalous El Niño storm events that heavily impacted aquatic habitat in the canyon. East Las Virgenes and Aliso were each represented by a single time series; the former was a postburn sample collected in the wake of the Topanga Fire, and the latter was collected after the Crown Fire but during the Station Fire.

### Microsatellite development

We isolated microsatellite loci from genomic DNA using a modification of the Hamilton et al. (1999) protocol that forgoes cloning and instead uses 454 technology to sequence loci directly from enriched libraries. The library was constructed by excising genomic DNA into different fragment sizes using *Hinc*II, followed by ligation of an SNX linker to these fragments. Biotinylated probes that included di‐, tri‐, and tetranucleotide repeats were then used to isolate and separate microsatellite loci from the rest of the DNA extraction. These fragments were PCR amplified and sequenced on a 454‐automated pyrosequencer (Roche) by the Evolutionary Genetics Core Facility (EGCF) at Cornell University.

From an initial pool of 54 loci, we identified 15 that amplified consistently and were variable across sampling locations (primer sequences are provided in Table S1). We divided these loci into four groups and performed multiplex PCRs using a Qiagen Multiplex PCR Kit^®^. All genotyping runs were performed on an ABI 3730 in the CSUPERB Microchemical Core Facility at San Diego State University. We edited and scored the raw data in gene‐marker v1.90 (SoftGenetics) and used microchecker (Van Oosterhout et al. 2004) to screen for null alleles. We tested for linkage disequilibrium (LD) by population using genepop on the web (Raymond and Rousset 1995).

### Inferring population structure

We used assignment methods in structure v2.3 (Pritchard et al. 2000) to identify clusters and spatial clines in allele frequencies. Cluster membership was assigned by specifying a range for the maximum number of clusters (*K *=* *2–10) to which individuals could be assigned. For each *K*, we performed 10 separate runs (250 000 steps; burn‐in 100 000) using both the correlated and uncorrelated allele frequency models with admixture. To approximate the number of clusters, we used structure harvester (Earl and vonHoldt 2011) to calculate Δ*K* (Evanno et al. 2005) and plotted the mean lnP(D|*K*) score for each of the 10 runs against *K*, where the number of clusters was based on the asymptote of the lnP(D|*K*) curve. Based on these results, we performed a second round of assignment tests on clusters from the initial analysis that included multiple sampling locations (following Coulon et al. 2008). The goal of this approach was to detect finer geographic structuring that may be more reflective of a true population, and to use these results as the units for estimating population diversity.

For all structure analyses, we generated alignments of the assignment coefficient matrices across independent runs using the GREEDY algorithm in clumpP v1.1.2 (Jakobsson and Rosenberg 2007). Optimal alignments for a range of *K*_MAX_ were then visualized using distruct v1.1 (Rosenberg 2004).

We sequenced 1013‐bp of the mitochondrial cytochrome *b* gene (*cytb*) for two individuals in each population and analyzed the phylogeography of haplotypes across the same geographic area sampled for microsatellites. We used the primers MVZ15‐L (Moritz et al. 1992) and CytbAR‐H (Goebel et al. 1999) to ensure that our sequences would overlap with those of previous studies on *R. draytonii* (e.g., Shaffer et al. 2004; Pauley et al. 2008). Bayesian Information Criterion (BIC) scores were calculated in *jmodeltest* 2.1.2 (Posada 2008) to identify the most appropriate model of evolution, and phylogenetic analyses were performed in BEAST v1.61 (Drummond and Rambaut 2007). We ran the BEAST analysis for 20 000,000 iterations, sampling from the posterior every 1000th iteration. We discarded the first 2000 samples and assessed convergence and effective sample sizes in tracer v1.5 (Drummond and Rambaut 2007). A maximum clade credibility tree was used to summarize the 95% credible set of trees.

### Genetic diversity

We performed a series of analyses to compare center‐edge trends in genetic diversity and relatedness. We measured observed heterozygosity (*H*_O_), expected heterozygosity (*H*_E_), Queller and Goodnight's relatedness index (*R*), and the inbreeding coefficient *F*_IS_ in genalex v6.0 (Peakall and Smouse 2006) and used a rarefaction procedure to estimate allelic richness *A*_R_ in fstat (Goudet 2001). To test for relationships between sampling location and genetic diversity, we performed separate linear regression analyses in R (R Development Core Team 2008) using *A*_R_, *H*_O_, and *R* as dependent variables and latitude and longitude as independent variables.

We estimated effective population sizes (*N*_e_) using approximate Bayesian computation (ABC) in OneSAMP (Tallmon et al. 2008) and the linkage disequilibrium (LD) method in LDne (Waples and Do 2008). For ABC estimates (hereafter *N*_e(ABC)_), we specified a noninformative, flat prior on *N*_e_ (5–1000) based on *N*_e_ estimates across a range of amphibian taxa, including several North American and European ranids (reviewed in Schmeller and Merilä 2007; Phillipsen et al. 2011). We performed replicate analyses and experimented with different priors to evaluate consistency in our results (Supplemental Information). For LDne analyses, we enforced a minimum allele frequency of 0.02–0.05 depending on sample size (Waples and Do 2010) and used a jackknifing procedure to calculate confidence intervals. We also implemented the temporal method (*N*_e(TM)_; Waples 1989) to provide a comparative *N*_e_ estimate for the San Francisquito population, given that we had three samples separated by multiple generations.

We used two approaches to infer population bottlenecks. First, we tested for heterozygote excess using a two‐phase mutation model and a Wilcoxon signed‐rank test in bottleneck (Cornuet and Luikart 1996). Second, we used the *M*‐ratio approach implemented in M_P_VAL (Garza and Williamson 2001); *M*‐ratios for each population were calculated using polymorphic loci only. We assessed significance by calculating a critical value, *M*_c_ relative to the equilibrium distribution through simulations in critical_M (Garza and Williamson 2001). Evidence for a significant reduction in population size is demonstrated if less than 5% of the simulated data sets (*n = *10 000) are below the observed *M‐*ratio. We also considered low allelic richness, low heterozygosity, and increased numbers of monomorphic loci to be an indicator of a bottlenecked population, as excess heterozygosity diminishes over time if the population size remains small.

Having detected bottlenecks in the three fire‐affected edge populations (San Francisquito, Aliso, and East Las Virgenes), we used coalescent‐based simulations under different demographic scenarios to approximate the timing of major reductions in *N*_e_ for each population. Our approach consisted of two steps: (1) simulating data sets based on scenarios in which the historical effective size *N*_e1_ exceeded the current effective size *N*_e0_ at time *T* in the past (measured in number of generations); (2) estimating a posterior for *T* through an additional set of simulations by setting a prior based on the most likely scenario(s) from step (1). We inferred the ‘best‐fit’ scenarios from step 1 using the closest 10 000 simulated data sets and the logistic and direct approaches described in Cornuet et al. (2008); see also Beaumont et al. 2002; dependent variables consisted of mean values for the number of alleles, gene diversity, allele size variance, and the *M‐*ratio, all of which are sensitive to fluctuation in *N*_e_. For each of five scenarios, we set the *T*_n_ parameter to 10, 20, 50, 100, and 1000 generations, assuming that one of these scenarios would provide an approximation for the timing of bottlenecks. We placed uniform priors on *N*_e_ as follows: current effective size *N*_e0_ (5–50) and *N*_e1_ (51–10 000). Prior bounds for *N*_e0_ were based on the upper limits of the 95% CIs inferred for *N*_e(ABC)_ and *N*_e(LD)_, whereas the bounds for *N*_e1_ were restricted to values above 50 but were otherwise noninformative.

For Step 2, we used the same general model to estimate *T*_n_ and *N*_e_ as free parameters with uniform prior distributions. For San Francisquito, we implemented the same priors for *N*_e_ as described above and bounded the *T*_n_ prior (3–200) according to the number of generations separating our temporal groups (with 2009 representing *T*_0_, 2005–06 specified to be one generation in the past *T*_1_, and 2002 specified as two generations in the past *T*_2_). This forced the bottleneck to predate our earliest sample, which is reasonable given that the 2002 sample showed a significant bottleneck based on multiple techniques. For Aliso and East Las Virgenes, we expanded the lower prior bound for *T*_n_ to 1–200 because these sites were limited to single point samples. We estimated *T*_n_ using the closest 10 000 simulated data sets to the observed data based on the same summary statistics and regression approach as described above. All simulations were conducted in DIY‐ABC v1.0.4.46 (Cornuet et al. 2008), and code for running the different models is provided in the Data S1.

### Genetic differentiation

We estimated *F*_ST_ and *R*_ST_ for each pair of populations (Wier and Cockerham 1984; Rousset 1996) and tested for significant differentiation using an exact *G* test in genepop on the web. For pairwise analysis across the full sampling area, we used only the 2009 samples for San Francisquito. To quantify genetic isolation by distance, we used Isolation By Distance Web Service (IBDWS: Jensen et al. 2005) to conduct Mantel tests and reduced major axis regression based on pairwise *F*_ST_ and corresponding geographic distance matrices. We then compared the residuals among samples within clusters as identified in the structure analysis using a Kruskal–Wallis analysis of variance by ranks. This approach accounts for effects of isolation by distance while testing whether population differentiation increased toward the range edge. Because the three edge populations each formed exclusive clusters at *K*_MAX_ = 6, we treated these sites as a single ‘edge’ group for this comparative test.

To test for population differentiation in San Francisquito Canyon before and after the 2002 Copper Fire, we used a permutation test in fstat to compare estimates of allelic richness, *H*_O_, *H*_E_, and *F*_ST_ for the different temporal sampling groups (i.e., 2002, 2005–06, and 2009). We lumped the 2005–06 samples because of small sample sizes for those 2 years (*n *=* *15 and 8, respectively) and because both were obtained in the aftermath of debris flows that were directly caused by the Copper Fire. We also performed an analysis of molecular variance (amova) in arlequin v3.5 (Excoffier et al. 2005) to assess the degree to which sampling year could explain the total amount of genetic variation among the three temporal groups.

## Results

### Population structure and phylogeography

**Figure 2:**
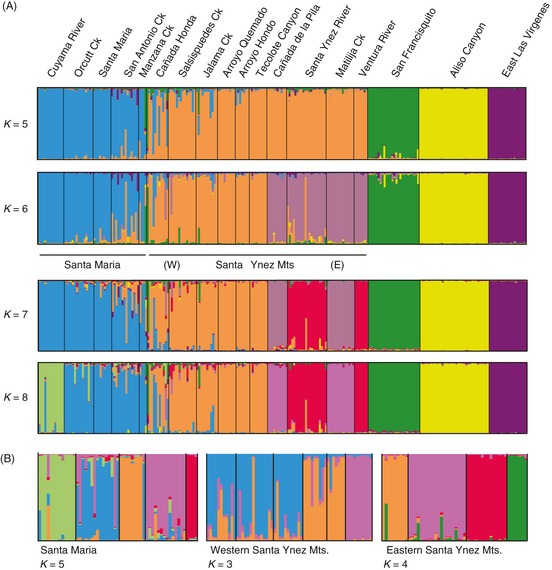
(A) Plots of the assignment coefficients estimated in structure (*K*_MAX_ **= 5–8). Populations are arranged in geographic order from the west/northwest part of the sampling range to east/southeast (left to right, respectively). (B) Nested structure analyses based on clusters with multiple‐site membership identified at *K*_MAX_ **= 6 (delimited by solid bars).

We found no qualitative differences in the cluster assignments based on models using correlated versus uncorrelated allele frequencies; thus, we discuss only the results from the uncorrelated analyses. Visual inspection of the lnP(D|*K*) curve and the Δ*K* statistic indicated six clusters as an estimate for *K*_MAX_, three of which were exclusive to individuals belonging to each range‐edge population (Fig. 2). Edge clusters showed no indication of admixture and were highly distinctive even at the lowest *K* value examined (although at *K *=* *2 the three edge populations formed a single cluster).

**Figure 3:**
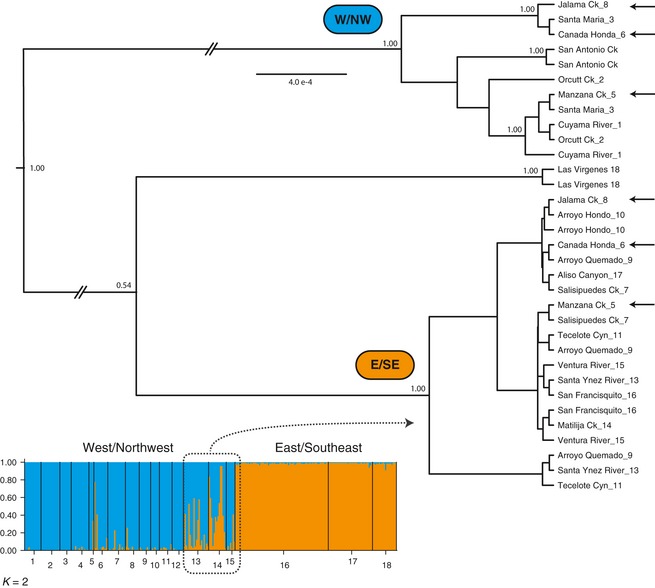
Assignment plot for *K *=* *2 and a maximum clade credibility tree based on mtDNA sequence data. Box overlaying the assignment plot shows that populations with admixed microsatellites alleles are all members of the east/southeast phylogroup. Arrows at the tree tips depict sites with mixed‐clade mtDNA haplotypes. Numbers above the branches are posterior probabilities.

Of the remaining clusters identified at *K*_MAX_ = 6, there was strong concordance between geography of the sampling locations and the grouping of individuals (Fig. 2). One exception to this pattern was observed at Cañada de la Pila, a site where individuals did not cluster with nearest‐neighbor populations in adjacent drainages less than 1.5 km away. We detected a clinal shift in allele frequencies near the west end of the Santa Ynez Mountains near Point Conception (San Antonio Creek, Cañada Honda and Salsipuedes Creek), with clusters roughly separating on either side of the mountains. This shift coincides geographically with a phylogeographic break in mtDNA haplotypes, with admixed individuals at Cañada Honda and other nearby sites having mixed‐clade membership (Fig. 3). At *K *≥* *8, the northernmost sample in the outer Coast Ranges (Cuyama River) formed an exclusive group, a pattern also recovered in the nested structure analyses. Nested cluster analyses revealed further evidence of population substructuring, with most sampling areas separating into distinctive clusters. Some exceptions occurred for groups of sites in adjacent drainages on the south‐facing slopes of the Santa Ynez Mountains and near Point Conception, all of which lie in close proximity and showed relatively low differentiation (Table S3).

Population structuring based on the microsatellites largely mirrored the diversity and distribution of mtDNA haplotypes (Fig. 3). We recovered six haplotypes with 13 informative sites, with one haplotype predominating the more southerly samples. We found strong support for two regional groups that corresponded to northwestern and southeastern sampling locations (Fig. 3). The northwestern group showed higher diversity and several well‐supported subdivisions, whereas the southeastern group showed no internal geographic structure. Three sites had frogs with mixed‐clade membership, corresponding to the same general area where individuals showed transitional admixture in microsatellite alleles. Furthest south, East Las Virgenes haplotypes were diverged from all others and were not found anywhere else in the sampling range, but their placement within the tree could not be resolved with strong statistical support (although a neighbor‐joining tree based on Cavalli‐Sforza chord distances of microsatellite data indicates that East Las Virgenes frogs have a most recent shared history with those in San Francisquito and Aliso Canyons; Fig. S1).

### Genetic diversity

All microsatellites conformed to mutational expectations as predicted by repeat motif, with the exception of allele 198 at RADR4‐09, which we determined was an out‐of‐phase indel (see Supplemental Materials for details). We detected possible null alleles and significant LD in two loci for the San Francisquito population only, but we suspect that these anomalies represent demographic artifacts (e.g., severe reduction in *N*_e_) rather than amplification or other sample‐wide problems. Table S2 provides motif identities and summary statistics for all loci.

Diversity measurements by cluster based on the nested structure analysis varied widely by location; *A*_r_ ranged from 1.79 to 3.48, *H*_O_ from 0.30 to 0.66, and *H*_E_ from 0.27 to 0.66 (Table 1). When considering only sites with >10 sampled individuals, East Las Virgenes and Aliso fell at the lower bound for each index. The highest diversity was detected near Point Conception and at sites further north. Pairwise relatedness of individuals varied in a manner predicted by the diversity indices; East Las Virgenes and Aliso frogs had the highest relatedness values (0.61 and 0.77, respectively), whereas the lowest values were found at Point Conception and Orcutt Creek (0.11 and 0.26; Table 1). Estimates of *A*_R_, *H*_O_, and *H*_E_ across year groups for San Francisquito (i.e., 2002, 2005–06, and 2009) revealed no significant changes in *A*_R_ (*P *=* *0.25; 1 × 10^4^ permutations), *H*_O_ (*P *=* *0.30), *H*_E_ (*P *=* *0.22), or relatedness (*P *=* *0.49) for pre‐ and postfire samples.

**Table 1:** Sample sizes and genetic diversity indices for clusters identified in the nested structure analyses

Sample site	*N*	*H*_O_	*H*_E_	*A*_R_	*R*	*N*_e(LD)_	*N*_e(ABC)_	*M*‐ratio	*H*_E_ excess	*A*_priv_	*A*_mono_
Cuyama River	13	0.40	0.41	2.23	0.20	10.4 (4.9–26.2)	9.63 (8.23–13.48)	0.69 (0.77)*		1	2
Orcutt Creek	15	0.62	0.61	3.23	0.14	28.7 (16.6–72.6)	19.13 (16.75–26.71)	0.83 (0.78)	**	0	0
Santa Maria	9	0.43	0.49	2.80	0.20	–	–	–		0	1
Manzana Creek	4	0.60	0.49	2.67	0.27	–	–	–		0	0
San Antonio Ck	14	0.63	0.60	3.16	0.10	66 (21.4–∞)	15.72 (13.35–23.04)	0.80 (0.78)	*	0	0
West Santa Ynez Mts.	35	0.66	0.66	3.48	0.05	132.2 (65.9–1113.1)	36.56 (32.63–56.47)	0.89 (0.80)	*	3	0
Central Santa Ynez Mts.	16	0.62	0.58	3.01	0.11	45 (21.3–510.2)	19.61 (17.41–26.19)	0.86 (0.78)	***	0	0
Tecolote Canyon	10	0.41	0.41	2.38	0.22	29 (8.4–∞)	14.21 (11.44–25.43)	0.90 (0.76)		0	1
Santa Ynez River	20	0.55	0.54	2.93	0.08	649 (43.8–∞)	28.89 (25.30–43.74)	0.79 (0.79)	*	4	0
Matilija Creek	13	0.49	0.46	2.43	0.23	13.8 (5.6–67.9)	15.73 (13.63–21.45)	0.83 (0.77)		0	0
Ventura River	7	0.33	0.36	2.15	0.22	–	–	–		0	2
San Francisquito 2009	26	0.61	0.54	2.53	0.20	11 (7.4–16.8)	20.27 (17.80–25.12)	0.75 (0.78)*	***	0	0
San Francisquito 2005–06	24	0.56	0.59	2.72	0.00	46 (21–525.5)	29.33 (24.49–47.54)	0.78 (0.76)	***	0	0
San Francisquito 2002	28	0.62	0.58	2.66	0.00	65.2 (25.9–∞)	30.93 (26.92–43.60)	0.76 (0.77)*	***	0	0
Aliso Canyon	35	0.36	0.38	2.17	0.25	18.1 (8.4–47.1)	29.73 (25.14–44.79)	0.73 (0.80)*	*	2	2
East Los Virgenes	19	0.30	0.27	1.79	0.34	12.3 (2.5–∞)	15.93 (13.45–21.51)	0.74 (0.78)*		0	2

Notations are as follows: *N*, sample size; *H*_O_, observed heterozygosity; *H*_E_, expected heterozygosity; *A*_R_, allelic richness; *R*, Queller and Goodnight's relatedness index; *N*_e(LD)_, effective population size based on linkage disequilibrium; *N*_e(ABC)_, effective population size based on approximate Bayesian computation; *M*_c_, Garza & Williamson's *M*‐ratio and its critical value; (*P *<* *0.05*; *P *<* *0.01**; *P *<* *0.001***), significance for *H*_E_ excess tests based on a Wilcoxon signed‐rank test; *A*_priv_, number of private alleles; and *A*_mono_, number of monomorphic alleles.

Sites with multiple sampling locations are the West Santa Ynez Mountains (Salsipuedes Cr., Cañada Honda and Jalama Cr.) and the Central Santa Ynez Mountains (Arroyo Quemado and Arroyo Hondo). We do not report *N*_e_ estimates or results of bottleneck tests at sites with fewer than 10 samples.

We found significant positive relationships between increased longitude of the sampling location and *A*_R_ (*r* ^2^ **= 0.60; β = 0.77; *P *<* *0.001) and *H*_O_ (*r* ^2^ **= 0.37; β = 0.60; *P *<* *0.008)_,_ and a significant negative relationship with respect to the average relatedness among individuals within a sample *R* (*r* ^2^ **= 0.52; β = −0.72; *P *<* *0.001). We did not detect any significant associations between these same indices and latitude. *N*_e(LD)_ estimates also suggested a longitudinal effect, where populations closer to the range edge tended to have smaller effective sizes (Table 1). However, values for several sites had wide confidence intervals, with some having an upper bound of infinity. ABC estimates were consistent with *N*_e(LD)_ values for some locations and independent onesamp runs produced very similar results (*r *=* *0.92); however, the ordinal geographic pattern was not upheld in the former. Both *N*_e(LD)_ and *N*_e(ABC)_ showed decreasing trends across sampling years for San Francisquito. Error estimates for both approaches were overlapping across year classes, indicating that any temporal signal in the data was weak. The *N*_e(TM)_ estimate for San Francisquito (30.92, 95%CI = 17.8–67.58) was in strong agreement with the ABC results for the 2002 and 2005–06 samples, and the slightly wider CIs also encompassed the *N*_e(LD)_values.

We detected excess heterozygosity in seven of the 14 clusters spread throughout the sampling range (Table 1), with many falling within or near fire footprints (Fig. 1). The strongest signals were detected in San Francisquito (2009) and in drainages on the south slopes of the Santa Ynez Mountains (Table 1). Of the three populations in which fire effects have been documented, Aliso and all year classes at San Francisquito showed evidence of significant heterozygote excess, while East Las Virgenes and San Francisquito (2002 and 2009) showed evidence of bottlenecks based on significant *M*‐ratios (Table 1).

**Figure 4:**
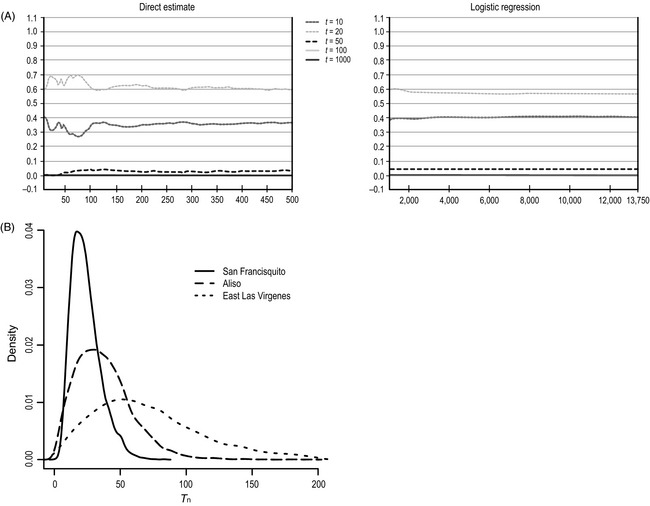
(A) Posterior probabilities (*y*‐axis) for the five scenarios using the direct and logistic approaches as reported from DIY‐ABC for San Francisquito Canyon. The *x*‐axis corresponds to the different *n*_δ_ closest data sets used in analyses. (B) Posterior estimates for the *T* parameter (generations before present) for the three range‐boundary populations. The value of *T* is the point in time where *N*_e_historical_ > *N*_e_pre‐fire contemporary_; thus, population declines would have occurred at some time after the estimated *T*_n_.

Comparison of competing demographic scenarios showed that the model in which *T* was fixed to 20 generations provided the closest fit to the observed data for San Francisquito and Aliso based on regression analyses (Fig. 4), whereas scenarios fixed to 50 and 100 generations provided closer fits for East Las Virgenes. When we estimated *T* as a free parameter with uniform prior probability, our results indicated that the reduction in *N*_e_ for East Las Virgenes was likely older (*T*_1_ mean = 70.8 generations; 95% CI = 15.2–149.7) than either the Aliso (*T*_1_ mean = 37.5; 8.4–76.3) or San Francisquito populations (*T*_1_ mean = 24.9; 9.5–44.95). These results were consistent with expectations based on the scenario comparisons from step 1. We note that the *T*_1_ parameter should be interpreted as the point in time in which the historical *N*_e_ was much larger than the current *N*_e_; thus, the actual population bottleneck would have occurred at some time after *T*_1_. Even when considering the lower bounds of the 95% CIs, these estimates indicate that major size reductions in each population predate the surge of recent fires in southern California.

### Genetic differentiation

Average pairwise values for *F*_ST_ and *R*_ST_ among sampling sites were 0.26 and 0.31, respectively, and overall among site differentiation was significant (*X* ^2^ = ∞, *P *<* *0.001). Pairwise *F*_ST_ and *R*_ST_ were significantly correlated based on a Mantel test (*r *=* *0.61, *P *=* *0.001); thus, we discuss only the *F*_ST_ estimates (range = 0.007–0.580; Table S3).

**Figure 5:**
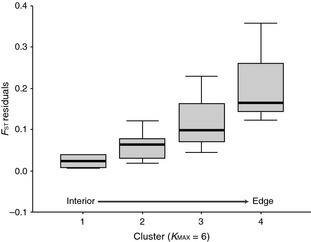
Comparison of residuals from the regression of pairwise *F*_ST_ values within clusters and corresponding geographic distances. Box plots reflect cluster assignments at *K*_MAX_ = 6, where clusters are arranged from the range interior to the edge (left to right). Cluster 4 is comprised of the three edge populations combined.

We detected a strong pattern of genetic isolation by distance (Z = 3.27 × 10^6^, *r *=* *0.55, *P *≤* *0.001) across the sampling range. Only a few sites along the southern slopes of the Santa Ynez Mountains and to the immediate west near Point Conception were not significantly differentiated. Differences among clusters identified at *K*_MAX_ = 6 showed that pairwise *F*_ST_ estimates for interior samples ranged from 0.09 to 0.19, whereas the average among the three edge populations was 0.31. Comparison of residuals from the regression of pairwise *F*_ST_ values and corresponding geographic distances confirmed that genetic differentiation among sites increased toward the range edge (χ^2^ = 11.22, df = 3, *P *=* *0.01; Fig. 5).

We conducted an additional set of pairwise *F*_ST_ comparisons across sampling years for San Francisquito. In contrast to the diversity estimates, we found significant differences among year groups sampled before and after the Copper Fire (*F*_ST_ = 0.035 over all loci among years; *P *<* *0.001, 1 × 10^4^ permutations), with larger differences observed as the sampling interval between pairwise comparisons increased (i.e., *F*_ST_ = 0.000 for 2002 vs 2005/06; *F*_ST_ = 0.023 for 2005/06 vs 2009; *F*_ST_ = 0.047 for 2002 vs 2009). Inspection of allele frequencies across year groups showed that five low frequency alleles from three loci completely dropped out of the population over the 7 year time gap following the Copper Fire (5/55 or ~9% of the total number of alleles across all years). Consistent with the comparison of diversity indices, the amova showed that only 2.1% of the total variation in pairwise *F*_ST_ estimates among year classes was explained by differences in the timing of the sampling.

## Discussion

Our study is one of a few examples of a rapid range contraction at low latitudes in a threatened amphibian species. The spatial configuration of *R. draytonii* populations forming the southern range edge and their interdigitation within major urban centers acts to suspend gene flow along the periphery of the distribution and raises conservation concerns for some of the most threatened populations in the species' range. On the one hand, the low diversity and isolation of sink populations at the range edge suggest that management efforts should focus on preserving high diversity, interior sources to the west/northwest (Hardie and Hutchings 2010). However, this ‘kick‐the‐can’ approach does little to halt the ongoing range contraction and may be especially detrimental if adaptive variation that enables population persistence in marginal environments, or even niche expansion, is completely purged from the species' gene pool (Vucetich and Waite 2003; Hampe and Petit 2005). It also begs the question as to how much range contraction is acceptable before any action should be taken to stop or reverse the process.

While this study does not address the adaptive costs of losing marginal *R. draytonii* populations, it does provide key insight about the genetic contributions to extinction debt at the range boundary, as well as an important framework for guiding translocation and genetic rescue efforts. Although the exact causes and their relative contributions to *R. draytonii* decline have yet to be resolved (but see Jennings and Hayes 1994; Fisher and Shaffer 1996; Davidson et al. 2001), the fact that such rapid extinction shows spatial and temporal concordance with some of the most expansive urbanization in all of North America leaves little doubt that the southern range front is receding largely as a consequence of human activity. This process may be augmented by the recent pace of climate change (also human‐mediated) and possibly historical climate‐related environmental shifts, which can also lead to ‘fraying’ at receding edges due to increased fragmentation and interpopulation differentiation (e.g., frogs of the *Rana pretiosa* complex; Green et al. 1996); however, the persistence of *R. draytonii* in more xeric habitats further south in Mexico, combined with similar edge decay in the northern part of the range in the Sierra Nevada (Fellers 2005), suggests that factors other than climate currently have an overriding effect in these recent extinctions.

### Center‐edge patterns of diversity

Although the generality of the abundant‐center model has been challenged (Sagarin and Gains 2002; Sagarin et al. 2006), our results are consistent with its genetic predictions in showing that intrapopulation diversity and the tendency for random mating within a site decline toward the range edge. A recent review of 134 studies involving 115 species (47 animal and 67 plant) demonstrated similar patterns by showing that on average, the number of cases displaying a reduction in intrapopulation diversity at the range margin was significantly different from random expectations (Eckert et al. 2008). In fact, frogs were singled out among all animal studies reviewed as having the highest number of examples in which this pattern was supported (85% of 13 studies).

The longitudinal trend in declining center‐to‐edge diversity in *R. draytonii* is attributable to the spatial orientation of populations along the Western Transverse Range, which constrains the directionality of gene flow to sites near the range boundary. The correlation between genetic diversity and edge proximity may reflect increased effects of drift and demographic collapses in at least some populations (Cornuet and Luikart 1996), several of which have been impacted by recent fire‐flood events. Of the three edge populations, Aliso and East Las Virgenes showed the clearest signs of distress, ranking lowest for *H*_O_, *H*_E_, and *A*_R_ across all sampling locations, and highest in average relatedness among individuals. The lack of any recent gene exchange and the inability for dispersal among these sites and San Francisquito Canyon suggests that diversity augmentation and translocation may be the only way to prevent or reverse the course of range contraction.

Center‐edge trends in effective size were less clear because of the inconsistency in *N*_e_ estimates using different analytical methods, and in some cases the wide error limits. These issues may relate to a lack information in the data for a given sample (Tallmon et al. 2008; Waples and Do 2010), with some sites simply having too few frogs to obtain reliable *N*_e_ estimates with 15 or fewer loci. Effective sizes in four closely related ranid frog species in the Pacific Northwest tend to be small (i.e., <50) and range from 20 to 30 individuals per population – for study sites with more than 15 samples, our results are in general agreement with these data and add to growing evidence that effective sizes for North American ranids are typically on the order of tens of individuals, rather than hundreds or thousands (Schmeller and Merilä 2007; Phillipsen et al. 2011). The *N*_e_ estimates for San Francisquito may be the most reliable given large sample sizes and the consistency between results from the widely used temporal estimator and the ABC method.

Mitochondrial haplotypes also supported a declining center‐edge trend in diversity; sites from Point Conception northward showed cross‐clade mixture, longer branch lengths, and greater geographic structure than those to the east/southeast at the range edge. Higher diversity in the north/northwest samples again speaks to human disturbance as a driving factor for range contraction in southern California, as climate effects (e.g., post‐Pleistocene glacial retreat or recent climate warming) that cause species to move poleward often leave older populations with higher genetic diversity at the more southern latitudes (Martin and McKay 2004; Howes and Lougheed 2008; Hardie and Hutchings 2010). In *R. draytonii*, a single haplotype predominates across the southernmost sampling locations, with the exception of East Las Virgenes, where frogs have a site‐specific haplotype that may signal the remnants of a third major group that formerly existed in southern California.

Increased diversity in populations sampled further away from the range edge is not only a consequence of greater connectivity at more interior sites, but also of secondary contact with gene flow between divergent *R. draytonii* groups on either side of the Santa Ynez Mountains, a phylogeographic division mirrored in a number of other co‐distributed vertebrate taxa (Fig. 3; see reviews in Calsbeek et al. 2003; Feldman and Spicer 2006; Rissler et al. 2006; Vandergast et al. 2008). The geography of these interior populations likely provides them with a key survival advantage over those at the range edge, as their spatial juxtaposition between divergent groups creates greater symmetry in the sources of genetic input and more frequent opportunities for migration. In fact, variation from both interior and more peripheral sources has a higher probability of entering these populations compared with those occurring toward the range edge.

### Center‐edge patterns of differentiation

A second prediction of the abundant‐center model is that genetic differentiation should increase with proximity to the species' boundary, reflecting greater isolation among edge populations. Our results showed that even after accounting for genetic isolation by distance, groups of populations occurring successively further away from the range boundary showed lower genetic differentiation and more instances of mixed cluster assignment than populations forming the current range edge (Fig. 5). This pattern is again consistent with the 134 case studies reviewed by Eckert et al. (2008) in showing that on average, among‐population differentiation increases from the center to range edge in a broad range of plant and animal taxa. Furthermore, of these 134 studies, increased differentiation often coincides with decreased levels of genetic diversity at the range edge.

This center‐edge trend also has significant management implications because greater isolation and differentiation can accelerate losses of diversity through drift, especially if populations are small and experience repeated bottlenecks. The current lack of a center‐edge connection prevents interior sources of genetic variation from ever reaching peripheral populations, where limiting environmental factors might impose greater challenges to survival and reproduction (Guo et al. 2005; Pearson et al. 2009; Sexton et al. 2009). Perhaps even more significant is the loss of interchange among edge populations themselves, as these connections may constitute more important sources of diversity at the range margin. Because the realized niche across the range edge is expected to be more similar than across a center‐edge transect, alleles evolving within the same niche are more likely to benefit neighboring edge populations, especially if the distances separating them are small (Sexton et al. 2011). Thus, diversity augmentation and translocation efforts in areas near the southern range boundary may have greater chances for success if sources are taken from the existing edge populations. This strategy would also preserve the historical phylogeographic structure of the species and minimize the chance of introducing maladaptive alleles from nonlocal areas (Kirkpatrick and Barton 1997; Edmands 2007).

Most sampling locations showed little evidence of recent gene flow, even in clusters with multiple sample site membership. This finding concurs with studies on other closely related ranid frogs, where populations tend to be highly structured by drainage basins (Funk et al. 2005; Lind et al. 2011; Schoville et al. 2011). Such structuring may be linked to the tendency for ridgelines and elevation gradients to restrict gene flow in montane frogs and salamanders (Funk et al. 2005; Spear et al. 2005; Giordano et al. 2007). Because dispersal ability is critical for maintaining diversity under range contraction, the limited interchange between even some of the more interior populations suggests that *R. draytonii* may be inherently slow to respond to conditions presented by a rapidly receding range front. However, Bulger et al. (2003) showed that *R. draytonii* are capable of moving considerable distances under normal circumstances (>3.5 km and up to 500 m away from water sources) and do so with little regard for topography or vegetation. Thus, when pressed under certain conditions, such as low density or the disappearance of habitat, these frogs may have a greater tendency to behave outside the norm.

### Processes giving rise to center‐edge patterns

USGS field survey data have shown dramatic demographic declines in *R. draytonii* edge populations following fires in southern California over the past decade. Observation of live adult frogs in the aftermath of fires and lag times between fire containment and the onset of population collapse suggests that burning or asphyxiation is not the primary cause of mortality. Rather, accelerated erosion, landslides, and debris flows during postfire rains appear to have the greatest impacts (Cannon et al. 2011; Parise and Cannon 2012). Specifically, landslides and fluvial debris cause direct mortality and displace aquatic habitat, which impedes breeding activity and precludes it altogether in extreme circumstances, such as during the El Niño storm events of 2005 (Snyder‐Velto 2008).

Historically, the fire‐flood sequence may not have had the same impacts in populations forming the range edge, as these now‐isolated headwater populations were replenishable by immigrants from lower reaches or other nearby sources. For example, San Francisquito and Aliso Canyons are tributaries of the upper Santa Clara River, and museum records show that *R. draytonii* were present along the upper Santa Clara main stem between these sites. Today, the distance between populations and the intervening urban areas prevents natural recolonization in the wake of catastrophic events, leading to greater risk of local extinction. This differs from fire‐affected sites located further away from the range edge (i.e., Manzana, Jalama, Arroyo Hondo, and Arroyo Quemado) where frogs are more abundant and occur in less disturbed habitat and therefore have greater opportunity to receive immigrants from outside of the effected areas. While several of the more interior populations showed evidence of demographic reduction, even a small number of immigrants from nearby sources could result in rapid genetic recovery during periods of low density (Keller et al. 2001; Busch et al. 2007), providing them with significant survival advantages over edge populations.

At the time scale examined here, we found no consistent genetic signals that were attributable to recent fire‐flood events, similar to studies on the genetics of recent population crashes in sea otters (Aguilar et al. 2008), kangaroo rats (Busch et al. 2007), and tigers (Henry et al. 2009). The pattern was most striking for San Francisquito Canyon, where the 2009 sample showed little genetic imprint of the population's collapse after the Copper Fire (2002) and El Niño storm events of 2005. Two main factors explain this pattern. First, rapid punctuated declines tend to have little impact on the amount of genetic diversity in the near term, with fast declines tending to preserve diversity better than slow, prolonged contractions (Leblois et al. 2006; Arenas 2011). Long generation time also creates inertia in this process (Kuo and Janzen 2003; Lippé et al. 2006) – at most, our San Francisquito samples spanned two generations, leaving little time for genetic drift to purge variation from the population. Second, demographic events extending to the deeper history of these edge populations are likely masking the effects of recent fires. *M‐*ratios fell below critical *M*_c_ values at all edge sites, including pre‐ and postfire samples for San Francisquito and two showed significant heterozygote excess (Table 1). Simulations also confirmed that the timing of reductions in *N*_e_ occurred well before the fire‐flood sequences over the past decade. If historical demographic fluctuations at these sites were severe enough, it is possible that genetic diversity estimates will leave little trace of more recent bouts of decline.

One condition under which this could happen is through rapid population expansion following a bottleneck, as might be expected after a founder event. In this case, subsequent periods of decline (or growth) may not erase the signature of the initial expansion unless the population once again approaches drift‐mutation equilibrium, or the secondary decline is severe and protracted (Lavery et al. 1996; Rogers 1997; Busch et al. 2007). Our simulations, combined with historical locality records for *R. draytonii* and local hydrology, suggest that an historical founder event followed by rapid growth is likely obscuring the genetic signals of the recent fire‐flood decline in the San Francisquito population. Assuming a generation time of three to 4 years for *R. draytonii* (Jennings and Hayes 1985) and a mean *T*_1_ of 24.9 generations from our simulations (Fig. 4), we estimate that this population was severely bottlenecked approximately 75–100 years ago. Remarkably, this decline coincides with the collapse of the St. Francis Dam in March of 1928, dubbed one of the greatest American civil engineering failures of the 20th century (Begnudelli and Sanders 2007). The dam's collapse in the mid to upper reaches of San Francisquito Canyon produced a floodwater surge of 45 million m^3^ and a break wave estimated at over 38 m high, scouring the canyon and the Santa Clara River floodplain as it travelled 87 km in 5.5 h to the Pacific Ocean.

Because the current breeding pools owe their existence to remnants of the dam's infrastructure and plunge pool, and museum records indicate that the canyon did not support *R. draytonii* prior to the dam's construction (the earliest records are from 1945: USNM123322‐23), we hypothesize that the bottleneck was caused by a founder event in San Francisquito Canyon, where frogs colonized the area from upstream reaches of the Santa Clara River that were unaffected by the disaster once habitat developed *in situ* after the dam collapsed. Under this scenario, our *T*_1_ and historical *N*_e_ estimates would reflect the status of this putative source population well before *R. draytonii* or the dam ever existed in San Francisquito Canyon.

### Implications for translocation and genetic rescue

We outline a strategy for selecting source populations to help fulfill translocation and genetic rescue efforts by the National Park Service and the U. S. Fish and Wildlife Service (Supplemental Materials). Our approach focuses on the ways in which the historical population structuring of *R. draytonii* can be maintained while simultaneously maximizing the amount of genetic diversity in rescue populations. Translocation sites in the Santa Monica Mountains of coastal California are currently under consideration because of the availability of protected lands and the former presence of these frogs throughout this area. Today, the only surviving *R. draytonii* population in the Santa Monica Mountains occurs in East Las Virgenes Canyon.

Our findings emphasize the need for continuous genetic monitoring over longer temporal scales following major contemporary disturbances to better understand the genetic consequences of these events, especially for species like *R. draytonii* with relatively long generation times. They also reveal the ways in which historical demographic events can increase extinction debt and limit the genetic resources of a population before more contemporary disturbances even occur – this problem may be particularly acute if the population is already naturally challenged due to its location at the edge of a species boundary.

## Data archiving statement

Microsatellite data for this study are available on the Dryad database (DOI:10.5061/dryad.g2b4h; www.datadryad.org), and DNA sequences are available on GenBank (accession #'s KC736503 – KC736550; www.ncbi.nilm.nih.gov/genbank/).

## Supporting information


**Figure S1.** Unrooted neighbor‐joining tree based on the pairwise Cavalli‐Sforza chord distances.
**Table S1.** Locus labels, primer sequences, fluorescent dyes, and multiplex PCR mixes for the 15 microsatellite sequences used in this study.
**Table S2.** Summary statistics for the microsatellites developed for *R. draytonii*.
**Table S3.** Pairwise *F*_ST_ values.
**Data S1.** Model codes for DIY‐ABC analyses.
