# Supplementary material for: Evolutionary dynamics of a rapidly receding southern range boundary in the threatened California Red‐Legged Frog (Rana draytonii)
Source: Evol Appl. 2013 Apr 3;6(5):808–22. doi: 10.1111/eva.12067 (PMC5779129; doi:10.1111/eva.12067)
Supplement: Supplementary file 1 — Figure S1. Unrooted neighbor‐joining tree based on the pairwise Cavalli‐Sforza chord distances. Table S1. Locus labels, primer sequences, fluorescent dyes, and multiplex PCR mixes for the 15 microsatellite sequences used in this study. Table S2. Summary statistics for the microsatellites developed for R. draytonii. Table S3. Pairwise F ST values. Data S1. Model codes for DIY‐ABC analyses. [file EVA-6-808-s001.docx]

Supplementary Materials

Richmond, J. Q., K. R. Barr, A. R. Backlin, A. G. Vandergast and R. N. Fisher. 2012. Evolutionary dynamics of a rapidly receding southern range boundary in the threatened California Red-legged Frog (*Rana draytonii*)

**Conservation management and re-establishment efforts**

The U. S. Fish and Wildlife Service recovery plan for *R. draytonii* lists augmentation of existing populations and reestablishment of extirpated populations as priority management actions for the species in southern California (Recovery Tasks 1.27-1.35; USFWS 2002). Of the many risk factors to consider with either action, two relating specifically to genetics are inbreeding and outbreeding depression. Inbreeding depression results from an increased frequency of offspring that are homozygous for the same recessive allele, whereas outbreeding depression develops from crosses between parents adapted to contrasting environments or with chromosomal differences. Both can reduce reproductive fitness, and avoiding either phenomenon requires careful consideration of candidate source populations for translocation (Edmands 2007; Weeks et al. 2011).

The Santa Monica Mountains have been targeted for establishing rescue populations due to the availability of protected lands and the fact that *R. draytonii* were once common across this area. Based on the evolutionary genetic framework provided by this study, we recommend a translocation strategy that seeds rescue populations from sources that are consistent with the phylogeography of a given area, rather than randomly drawing samples from across the range or specifically from populations with the highest genetic diversity, regardless of location. This approach minimizes the potential for introducing nonlocal variation that might potentially be maladaptive at the range edge (Ficetola and De Bernardi 2005; Edmands 2007; Frankham et al. 2011), and maintains the natural population structure of *R. draytonii* in southern California.

For prospective rescue populations in the Santa Monica Mountains, the obvious source would be East Las Virgenes, given the strong pattern of genetic isolation by distance in our dataset and the likely historical interconnection of populations within this same geophysical area. Individuals might be translocated with little hesitation if not for the low diversity and small *N*_e_ of the current East Las Virgenes population, which raises concern about inbreeding depression. One way to circumvent this issue, however, would be to augment the rescue sample with small numbers of egg masses from the next closest sites, Aliso and San Francisquito Canyons. Just a few transplants from these alternative sources could have positive fitness benefits (Tallmon et al. 2004), and would also preserve population structure in a manner consistent with the evolutionary history of the species. By creating a conglomerate rescue population according to this strategy, one would be capturing the most genetic variation among populations within the same southern phylogroup, while simultaneously preventing the mixture of alleles from divergent and potentially adaptively differentiated populations.

Considering that *R. draytonii* on either side of the main phylogeographic break are notably divergent, outbreeding depression may be of concern if ‘northern frogs’ are intermixed with frogs that have evolved within the southern genetic background. Local adaptations could be lost and unforeseen genetic incompatibilities may result in the ensuing generations, reducing the likelihood of successful population establishment – this same concern has been raised in translocation considerations for the agile frog *Rana latastei* across elevation gradients, where genetically fixed differences in the timing of metamorphosis between high and low elevation populations are known to be adaptive (Ficetola and De Bernardi 2005). Although we are not aware of any obvious adaptive differences between the two *R. draytonii* phylogroups in this study, our approach recognizes the implications of the deeper history of isolation among populations at opposite ends of the Santa Ynez Mountains, preserves the historical integrity of the species in the southern-most part of its range in California, and at the same time captures as much of the remaining genetic variation as possible.

Literature Cited

Edmands, S. 2007. Between a rock and a hard place: evaluating the relative risks of inbreeding and outbreeding for conservation and management. Molecular Ecology 16:463-475.

Ficetola, G. F., and F. De Bernardi. 2005. Supplementation or in situ conservation? Evidence of local adaptation in the Italian agile frog *Rana latastei* and consequences for the management of populations. Animal Conservation 8:33-40.

Frankham, R., J. D. Ballou, M. D. B. Eldridge, R. C. Lacy, K. Ralls, M. R. Dudash, and C. B. Fenster. 2011. Predicting the probability of outbreeding depression. Conservation Biology 25:465-475.

Tallmon, D.A., Luikart, G., Waples, R.S., 2004. The alluring simplicity and complex reality of genetic rescue. Trends in Ecology & Evolution 19, 489–496.

**Supplementary tables**

**Table S1**. Locus labels, primer sequences, fluorescent dyes, and multiplex PCR mixes for the 15 microsatellite sequences used in this study. A GTTTCTT ‘pigtail’ was added to the 5’ end of the reverse primer to reduce stuttering for some loci.

| Locus | Forward | Reverse | Dye | MP Mix |
| --- | --- | --- | --- | --- |
| RADR4-03 | CAACCACACAGCCATTAGGA | TTGACCAATAGGTTACACTTCTGC | VIC | 1 |
| RADR3-01 | CTTGAGGGCAGGGACTGAT | GGCAAAACTGGGAGACATTG | NED | 1 |
| RADR4-01 | ACACGAGGCTGAAAGGAATG | AATCAAGGCGTTGGGATAGA | FAM | 1 |
| RADR4-02 | CATTGGTGGGAAGCATTTCT | TGGATTTTTCAACATGACAATGA | FAM | 1 |
| RADR4-05 | CCATCCATTTCTCCCTACTTTG | TGGATGAATGCTGCTACGAG | PET | 2 |
| RADR4-04 | TGAGAGCGACCCATTCTTTC | CTGGGGTAGTGCTACAGATGG | NED | 2 |
| RADR3-02 | GCTGCCAACTGACATTGAGA | TGGTGTGGAGACCAGCATAA | PET | 2 |
| RADR4-06 | GTTTCTTCAGACAGATATGAAATAAGCACAAATG | AGCTACAACAAACGTAAAAGTATC | NED | 3 |
| RADR4-07 | GGTTGGCGGAGCTGTACTC | GTTTCTTTGTGTTCTGTTGCGTACCTTG | FAM | 3 |
| RADR4-08 | CGATGGTCAAACAGGGAGAC | GTTTCTTAGGTTTTCTACATATCACTCAATAAGC | VIC | 3 |
| RADR4-09 | CCCCACACCTAATGTCCAAG | GTTTCTTTCACTATGGCTACCCTTCACG | PET | 3 |
| RADR4-10 | ATAAAGCCACAGCCCAAGG | GTTTCTTTCCCTCAAAGCCTCAAAGAC | NED | 4 |
| RADR4-11 | TGGTTTGGCTGTCATTTATCTG | GTTTCTTGGTTGATAGATAATTAGGTAATCGATAG | FAM | 4 |
| RADR4-12 | GTTTAACATTCTACCATAGATTGAG | GTTTCTTTATAAGCTCCAAACAATTAAATCTTTAC | VIC | 4 |
| RADR4-13 | ATGGATATACTGTAGACAGATGG | GTTTCTTAGATACACTTCCAGGTACTCC | PET | 4 |

**Data quality**

All 15 microsatellite loci conformed to mutational expectations as predicted by repeat motif, with the exception of a single allele (198) at RADR4-09 in some individuals. We re-analyzed these individuals using a different *Taq* polymerase and recovered the same allele in a second round of genotyping. Thus, we considered 198 to be a real allele with an out-of-phase indel.

We detected possible null alleles only in the San Francisquito population: locus RADR4-10 was suspect when all sampling-year groups were combined; locus RADR4-05 was suspect in the 2002 sample; and locus RADR4-11 was suspect in the 2009 sample (Table 2). We also detected significant LD between two loci (RADR3-02 and RADR4-08; *P* <0.0001) in the 2009 sample. Additional analyses described in the text show that San Francisquito population has undergone a severe reduction in *N_e_*, in which case deviations from Hardy-Weinberg equilibrium would be expected, and none of the other populations showed evidence of null alleles across the 15 loci. Thus, we suspect that demographic artifacts of this population rather than amplification or other sample-wide problems caused the null allele signal.

**Table S2.** Summary statistics for the microsatellites developed for *R. draytonii*. Notations are as follows: total number of alleles *A,* allelic richness *A*_r_, observed heterozygosity *H*_O_, and expected heterozygosity *H*_E_. The library was constructed using DNA from an individual sampled in San Francisquito Canyon.

| Locus | Motif | Size Range | *A* | *A*_R_ | *H*_O_ | *H*_E_ |
| --- | --- | --- | --- | --- | --- | --- |
| RADR3-01 | (ATC)^7^ | 209-223 | 4 | 2.556 | 0.475 | 0.454 |
| RADR3-02 | (GAG)^9^ | 112-115 | 2 | 1.889 | 0.346 | 0.325 |
| RADR4-01 | (ATCT)^12^ | 174-190 | 5 | 3.056 | 0.500 | 0.515 |
| RADR4-02 | (ATCT)^7^ | 243-279 | 10 | 4.722 | 0.664 | 0.659 |
| RADR4-03 | (ATCT)^10^ | 186-206 | 6 | 3.278 | 0.505 | 0.479 |
| RADR4-04 | (ATCT)^8^ | 202-234 | 7 | 3.111 | 0.458 | 0.444 |
| RADR4-05 | (ATCC)^5^ | 162-186 | 9 | 3.611 | 0.485 | 0.460 |
| RADR4-06 | (AGAT)^8^ | 138-150 | 4 | 2.611 | 0.329 | 0.352 |
| RADR4-07 | (AGAT)^13^ | 129-165 | 7 | 3.500 | 0.577 | 0.527 |
| RADR4-08 | (AGAT)^7^ | 128-146 | 3 | 2.389 | 0.407 | 0.396 |
| RADR4-09 | (AGAT)^10^ | 132-172 | 9 | 3.722 | 0.608 | 0.555 |
| RADR4-10 | (AGAT)^13^ | 172-220 | 13 | 5.278 | 0.679 | 0.649 |
| RADR4-11 | (AGAT)^10^ | 146-174 | 8 | 3.333 | 0.502 | 0.484 |
| RADR4-12 | (AGAT)^8^ | 188-220 | 9 | 4.500 | 0.734 | 0.641 |
| RADR4-13 | (AGAT)^19^ | 108-152 | 12 | 4.722 | 0.715 | 0.637 |

**Table S3**. Pairwise *F*_ST_ values. Bold values are significantly differentiated using an Exact *G* test; α = 0.05 adjusted using sequential Bonferroni correction.

|  | Cuyama River | Orcutt Creek | Santa Maria | San Antonio Creek VAFB | Manzana Creek | Cañada Honda Creek | Salsispuedes Creek | Jalama Creek | Arroyo Quemado | Arroyo Hondo | Cañada de la Pila | Tecolote Canyon | Santa Ynez River | Matilija Creek | Ventura River | San Francisquito Cyn | Aliso Canyon |
| --- | --- | --- | --- | --- | --- | --- | --- | --- | --- | --- | --- | --- | --- | --- | --- | --- | --- |
| Orcutt Creek | **0.15** |  |  |  |  |  |  |  |  |  |  |  |  |  |  |  |  |
| Santa Maria | **0.23** | **0.11** |  |  |  |  |  |  |  |  |  |  |  |  |  |  |  |
| San Antonio Creek VAFB | **0.19** | **0.08** | **0.13** |  |  |  |  |  |  |  |  |  |  |  |  |  |  |
| Manzana Creek | **0.32** | **0.16** | **0.27** | **0.19** |  |  |  |  |  |  |  |  |  |  |  |  |  |
| Cañada Honda Creek | **0.24** | **0.10** | **0.12** | **0.04** | **0.14** |  |  |  |  |  |  |  |  |  |  |  |  |
| Salsispuedes Creek | **0.28** | **0.15** | **0.18** | **0.11** | **0.20** | 0.01 |  |  |  |  |  |  |  |  |  |  |  |
| Jalama Creek | **0.32** | **0.16** | **0.18** | **0.10** | **0.22** | **0.01** | 0.01 |  |  |  |  |  |  |  |  |  |  |
| Arroyo Quemado | **0.36** | **0.23** | **0.26** | **0.17** | **0.25** | **0.07** | **0.07** | **0.08** |  |  |  |  |  |  |  |  |  |
| Arroyo Hondo | **0.31** | **0.19** | **0.21** | **0.13** | **0.24** | **0.06** | **0.07** | 0.08 | 0.03 |  |  |  |  |  |  |  |  |
| Cañada de la Pila | **0.42** | **0.28** | **0.33** | **0.24** | **0.35** | **0.17** | **0.18** | **0.18** | **0.17** | **0.21** |  |  |  |  |  |  |  |
| Tecolote Canyon | **0.38** | **0.25** | **0.30** | **0.19** | **0.29** | **0.11** | **0.11** | **0.12** | 0.03 | 0.08 | **0.23** |  |  |  |  |  |  |
| Santa Ynez River | **0.36** | **0.25** | **0.29** | **0.21** | **0.28** | **0.10** | **0.09** | **0.10** | **0.12** | **0.16** | **0.17** | **0.15** |  |  |  |  |  |
| Matilija Creek | **0.47** | **0.30** | **0.36** | **0.29** | **0.37** | **0.20** | **0.18** | **0.19** | **0.23** | **0.24** | **0.22** | **0.26** | **0.18** |  |  |  |  |
| Ventura River | **0.48** | **0.34** | **0.39** | **0.29** | **0.40** | **0.19** | **0.17** | **0.18** | **0.18** | **0.25** | **0.24** | **0.27** | **0.14** | **0.30** |  |  |  |
| San Francisquito Cyn | **0.32** | **0.24** | **0.24** | **0.20** | **0.22** | **0.10** | **0.13** | **0.14** | **0.17** | **0.18** | **0.23** | **0.23** | **0.16** | **0.22** | **0.24** |  |  |
| Aliso Canyon | **0.53** | **0.39** | **0.48** | **0.39** | **0.44** | **0.35** | **0.36** | **0.34** | **0.41** | **0.43** | **0.46** | **0.45** | **0.37** | **0.38** | **0.47** | **0.25** |  |
| East Las Virgenes Creek | **0.58** | **0.46** | **0.52** | **0.39** | **0.55** | **0.34** | **0.35** | **0.36** | **0.43** | **0.43** | **0.50** | **0.46** | **0.36** | **0.50** | **0.49** | **0.31** | **0.53** |

**Figure S1**. Unrooted neighbor-joining tree based on the based on pairwise Cavalli-Sforza chord distances. Cluster labels are provided for assignments at *K*_MAX_ = 6.


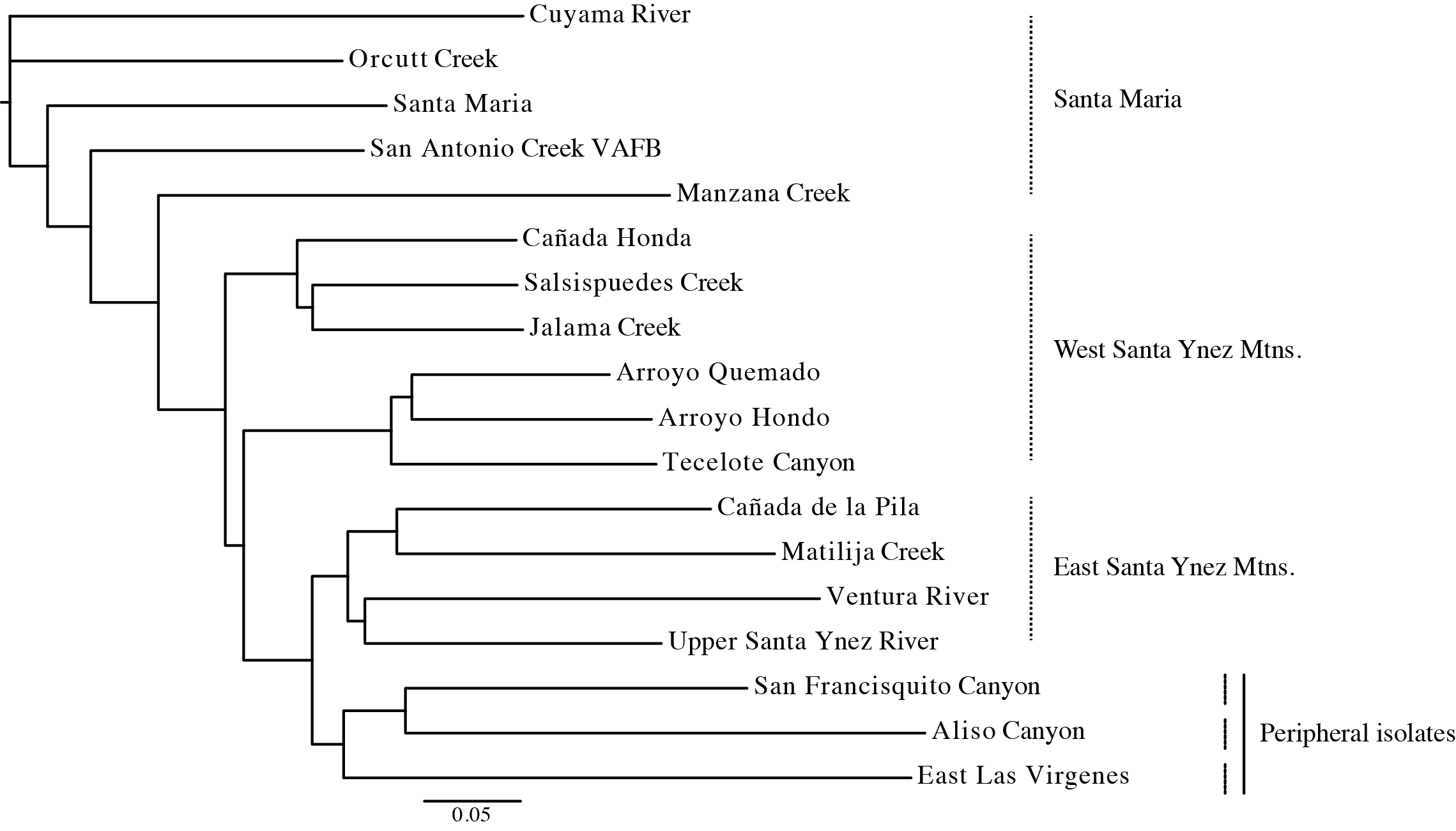


**Model codes for DIY-ABC analyses**

raau_san fran by yearg.gen # file name

header : 53 lines, 0 simulated datasets with 5 parameters and 12 summary statistics

5 scenarios

scenario 1 [0.2] (5) #description of each scenario compared

N0 #current effective population size; allowed to vary in the range listed below

0 sample 1 #tells DIY-ABC when in time the sample was taken; 0 = most recent, 1 = 1 generation ago

1 sample 1

2 sample 1

1000 varNe 1 N1 #1000 generations ago, the population size was N1 (allowed to vary in the range listed below)

scenario 2 [0.2] (5)

N0

0 sample 1

1 sample 1

2 sample 1

100 varNe 1 N1

scenario 3 [0.2] (5)

N0

0 sample 1

1 sample 1

2 sample 1

50 varNe 1 N1

scenario 4 [0.2] (5)

N0

0 sample 1

1 sample 1

2 sample 1

20 varNe 1 N1

scenario 5 [0.2] (5)

N0

0 sample 1

1 sample 1

2 sample 1

10 varNe 1 N1

N0 UN[5,50,0,0,1] #prior distribution for N0 in all scenarios (5 - 50)

N1 UN[51,10000,0,0,1] #prior distribution for N1 in all scenarios (51 - 10000)

Autosomal diploid microsatellites

MEANMU UN[1.00E-004,1.00E-003,-9,-9] #prior distribution for overall mutation rates

GAMMU GA[1.00E-005,1.00E-002,-9.00E+000,2.000] #individual locus mutation distribution shape (gamma), range (1e-5 - 1e-2), and shape (2)

MEANP UN[0.100,0.30,-9.00,-9.00] #overall distribution shape (gamma) and range (1e-1 - 3e-1) for P, which designates the Generalized Stepwise Mutation Model over the Stepwise Mutation Model

GAMP GA[1.00E-002,9.00E-001,-9.00E+000,2.000] #individual locus P values

MEANSNI LU[1.00E-008,1.00E-004,-9,-9] #overall INDEL mutation rate prior

GAMSNI GA[1.00E-009,1.00E-003,-9.00E+000,2.000] #priors INDEL mutations for individual loci

4 4 4 3 4 4 4 4 4 4 4 4 4 4 4 #repeat motif of each locus

80 80 80 60 80 4 80 80 80 80 80 80 80 80 80 #allele size range variation

Autosomal diploid microsatellites

NAL 1 2 3 #summary statistics used for finding closest pseudo-datasets (eg, NAL = no. of alleles)

HET 1 2 3

VAR 1 2 3

MGW 1 2 3

# used by DIY for summarizing psuedo-datasets

scenario N0 N1 µmic_A Pmic_A µ_SNImic_A NAL_1_A NAL_2_A NAL_3_A HET_1_A HET_2_A HET_3_A VAR_1_A VAR_2_A VAR_3_A MGW_1_A MGW_2_A MGW_3_A
